# 3D Markov Process for Traffic Flow Prediction in Real-Time

**DOI:** 10.3390/s16020147

**Published:** 2016-01-25

**Authors:** Eunjeong Ko, Jinyoung Ahn, Eun Yi Kim

**Affiliations:** Visual Information Processing Laboratory, Department of Internet & Multimedia Engineering, Konkuk University, Seoul 05029, Korea; goejeong85@gmail.com (E.K.); jyahn0515@gmail.com (J.A.)

**Keywords:** traffic flow forecasting, Markov process, spatio-temporal domain, vehicle detection sensor, heat map

## Abstract

Recently, the correct estimation of traffic flow has begun to be considered an essential component in intelligent transportation systems. In this paper, a new statistical method to predict traffic flows using time series analyses and geometric correlations is proposed. The novelty of the proposed method is two-fold: (1) a 3D heat map is designed to describe the traffic conditions between roads, which can effectively represent the correlations between spatially- and temporally-adjacent traffic states; and (2) the relationship between the adjacent roads on the spatiotemporal domain is represented by cliques in MRF and the clique parameters are obtained by example-based learning. In order to assess the validity of the proposed method, it is tested using data from expressway traffic that are provided by the Korean Expressway Corporation, and the performance of the proposed method is compared with existing approaches. The results demonstrate that the proposed method can predict traffic conditions with an accuracy of 85%, and this accuracy can be improved further.

## 1. Introduction

Accurate traffic flow prediction is receiving significant attention in the research of Intelligent Transportation Systems (ITSs) [1,2,3,4,5,6,7,8,9,10,11,12,13,14,15,16,17,18,19,20,21,22,23,24]. The short- and long-term traffic forecasting, *i.e.*, to provide the traffic flows of the next or several periods of time in the future, using real-time data is essential for providing traffic control in ITSs. Through traffic flow prediction, ITSs can control and manage traffic conditions.

During the past few decades, various algorithms have been proposed, and these are categorized into parametric and non-parametric approaches. Parametric approaches have been well established using mathematical models to describe the traffic state and its trends; thus, these approaches are also referred to as model-driven approaches. Numerous parametric methods have been investigated, including the simplest method using historical averages. Among these parametric methods, the most representative ones have been developed by Wang and Papageorgiou [2] and Ramezani *et al.* [3].

In earlier studies [2,4], Kalman filtering has been widely used for traffic state estimation, and it identifies the state model that is used to describe the evolution of traffic flows from historical traffic data. In addition, Gaussian mixture models (GMMs) have also been widely used in modeling traffic flows, where the processes of predicting traffic flows are formulated using the maximum likelihood estimation (MLE) framework and the model parameters of GMM are found through using a distributed expectation–maximization (EM) algorithm [3]. Recently, various models have been proposed to predict traffic flows from different environments including highways and urban roads, e.g., seasonal autoregressive integrated moving average (SARIMA) [5], multivariate spatio-temporal autoregressive (MSTAR), and cascade models [6]. Williams *et al.* proposed SARIMA for traffic flow forecasting, where the Wold decomposition is used for stationary transformation of discrete–time conditional traffic data and the Schwarz Bayesian information criterion (SBC) used as the optimal criterion [5]. Min and Wynter proposed a real-time traffic prediction method using the MSTAR model, where the model parameters are used to estimate the speed and volume of the traffic [6]. In addition, they also presented regime–switching space–time models that manage the nonlinearity and potential collinearity of the traffic data [7], where the model parameters, such as coefficients between neighbors, are estimated using the adaptive least absolute shrinkage and selection operator (LASSO). Sun and Zhang developed the selective random subspace predictor (SRSP) that constructs the selective input space using Pearson correlation coefficients and utilizes GMM with competitive expectation maximization (CEM) algorithm to derive the prediction formulation of traffic flows [8]. Sun and Xu proposed the infinite mixtures of Gaussian processes (IMGP) using the Dirichlet process prior, and variational inference techniques to predict the traffic flows of the urban roads [9]. In addition, Sun extended the IMGP to multivariate Gaussian processes to apply that model to large scale data [10]. Gao *et al.* employed graphical lasso to represent a relationship between links and estimate coefficients, and then utilized a neural network to approximate arbitrary bounded and continuous functions [11]. Piatkowski *et al.* proposed generative graphical models using a Markov random field (MRF) to represent complex relations among roads and consider all possible relations on the spatiotemporal domain using belief propagation, and estimated traffic flow states using maximum *a posterior* estimation (MAP) [12]. They applied the proposed graphical model with Gaussian process regression to estimate traffic flow in areas with low sensor coverage [13]. Short-term traffic flow forecasting was proposed based on time-varying conditional variance modeling of the traffic flow series: the seasonal ARIMA was combined with the generalized autoregressive conditional heteroskedasticity (GARCH) to predict both the traffic flow levels and conditional variances [4].

In contrast, non-parametric approaches are data-driven methods that do not require mathematical models [14,15,16,17]. The neural network (NN) and *K*-nearest neighbor (*K*-NN) are representative non-parametric approaches. *K*-NN uses the nearest neighbors from large historical data that were collected at the same timestamp on different days [14,15,16]. Gong and Wang defined the trends of traffic flows over several time periods as patterns and attempted to automatically locate similar patterns to the current pattern from the historical data of different times in the past [15]. The experiments demonstrated the effectiveness of a NN in accurately predicting traffic flows; however, it has significant drawbacks of requiring the selection of an appropriate learning dataset, as well as a complicated computational cost. Recently, Smith *et al.* proposed a short-term traffic flow estimation using the nearest neighbor non-parametric regression [17]. This non-parametric approach depends on the accuracy of the collected historical data; thus, it requires a large dataset. Furthermore, NN requires a more complicated training phase, unlike *K*-NN.

As illustrated above, most approaches in the literature have concentrated on analyzing the temporal correlation between the current and historical traffic flows [1,2,3,4,5,14,16,17,18,19,20,21,22,24]. Although the temporal correlation is important information in predicting the traffic state of the next time period, geometrical correlations should also be considered. In particular, adjacent roads influence each other’s traffic conditions; *i.e.*, if traffic congestion occurs on one road, its connected roads will have the same or similar traffic conditions after a period of time [6,7,8,9,10,11,12,13,15,23]. Therefore, we propose a novel method to predict traffic flows that uses both time series analyses and geometrical correlations. The novelty of the proposed method is two-fold: (1) a 3D heat map is designed to describe the traffic conditions between roads, which can effectively represent the correlations between spatially- and temporally-adjacent traffic states; and (2) the relationship between the adjacent roads on the spatiotemporal domain is represented by cliques in MRF and the clique parameters are obtained by example-based learning. 

The proposed system is composed of three modules: data preparation and filtering, 3D heat map modeling, and parameter estimation and prediction. The traffic data collected from expressways include numerous noises and errors; thus, we first conduct noise filtering and resampling using interpolation and statistics. Then, the heat map is constructed from the expressway data and it is modeled using the 3D Markov random field (MRF), which have been widely used to represent relations between adjacent roads in the spatio-temporal domain [12,13]. Based on the Markov process, traffic flow prediction is performed in which the spatial and temporal correlation parameters between adjacent roads are calculated using example-based learning. Using this model parameter, the prediction is performed through minimizing the energy function. 

In order to evaluate the effectiveness of the proposed method, it was tested with real traffic data provided by the Korean Expressway Cooperation (KEC). Then, the result was compared with an existing method. The result demonstrated that the proposed method can predict traffic conditions with an accuracy of 85% and that the proposed method can predict traffic conditions more accurately than the existing method.

## 2. Data Preparation and Filtering

The traffic data were provided by the Korean Expressway Corporation (KEC) (Korean Expressway Cooperation: [25,26]), which manages the traffic conditions of Korean expressways and analyzes the data. Among data from several expressways, we selected the traffic data from Gyeongbu expressway, because it has the largest traffic volumes and the main traffic zones. On Korean expressways, the basic unit of a road is called a cone zone. The Gyeongbu expressway consists of 1076 cone zones. For example, the expressway between the two cities of Gumi and Chilgok can be split into three cone zones: from Gumi Interchange (IC) to South Gumi IC, from South Gumi IC to Waegwan IC, and from Waegwan IC to Chilgok IC. 

In order to collect the traffic data from each cone zone, the KEC installed several vehicle detection sensors (VDSs) according to the length of the cone zone. The raw data includes the traffic condition observations at 30 s discrete intervals. The data consists of the average speed of the passing cars and the number of passing cars. The KEC have resampled the raw sensor data to 1 min intervals in order to analyze communication and predict unexpected accidents on the cone zones. Therefore, we also apply same intervals to the proposed method.

Due to data transmission and sensing problems, the raw data can include three types of noise: (1) missing data at a timestamp; (2) invalid data such as negative or excessively large numbers, e.g., up to 400 km/h; and (3) data that abruptly increases or decreases, usually occurring late at night and dawn. Thus, noise filtering should be conducted for accurate predictions.

In addition, the raw data can also contain zero numbers or numbers larger than the speed limit. These data are not noise; for example, the large numbers indicate free traffic conditions. The proposed method replaces these numbers with the value of the speed limit, e.g., 120 km/h. The zero numbers are considered to be congested or free according to the sensor data at the previous timestamp. Therefore, these numbers are also replaced by the nearest neighbor data.

For the purpose of our analyses, the proposed method first removes the noise from the raw data and then resamples the filtered data into 1 min. intervals. In order to remove the noise, the method separates the raw data according to the lane number. If the abovementioned noises are detected, those values are replaced by the nearest neighbor interpolation, e.g., the previous sensor value.

For the resampling stage, the filtered data for the separate lanes are combined using the harmonic average, as follows:

(1) $$v_{t}=\frac{n}{\frac{1}{x_{1}}+\frac{1}{x_{2}}+\ldots +\frac{1}{x_{n}}}=\frac{n}{\sum_{l=1}^{n}\frac{1}{x_{l}}}$$

where *n* is the number of lanes in one cone zone, $x_{l}$ is the average speed in lane *l* at time stamp *t*, $v_{t}$ is the combined speed of all lanes at time stamp *t*, and $y_{l}$ is the number of cars passing in lane *l*. In addition, the number of passed cars ($c_{t}$) is accumulated as described in the following equation:

(2) $$c_{t}=\sum_{l=1}^{n}y_{l}$$

The combined 30 s interval data are aggregated to 1 min. average values, which have been commonly used in the literature [2]. The equation for the aggregation is as follows:

(3) $$V_{m}=\frac{\sum_{t\in \left[m-1,\;m\right)\;}v_{t}*c_{t}}{\sum_{t\in \left[m-1,\;m\right)\;}c_{t}}$$

where *t* is the time index during one period, $v_{t}$ is the average speed, and $c_{t}$ is the number of cars passing at time *t*. The aggregated speed per 1 min. interval is averaged through multiplying the speed with the number of cars during one period.

**Figure 1:**
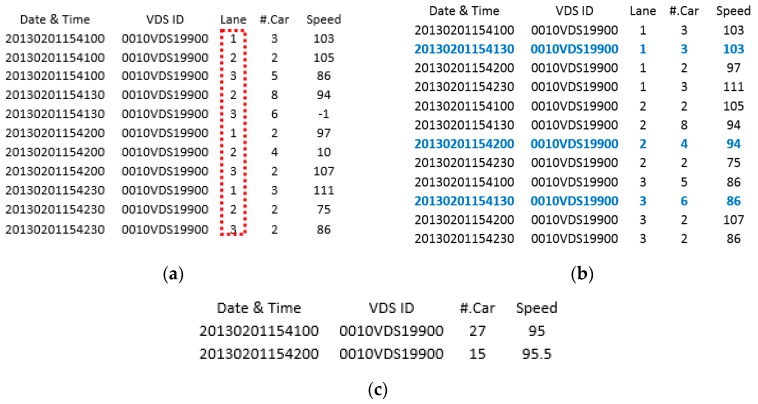
Data filtering and resampling results: (**a**) given raw sensor data; (**b**) noise filtered result; and (**c**) re-sampled result.

Figure 1 illustrates the process of noise filtering and resampling. Figure 1a presents the raw data that is sequentially collected from sensor “0010VDS19900” at every 30-s time interval. From left to right, the numbers correspond to the date and time, VDS ID, lane number, number of cars, and average speed. As seen in Figure 1a, the red rectangle indicates that the data is obtained from three different lanes in the cone zone. The proposed method separates the data according to the lane number and detects the noise. The blue fonts indicate data sorted according to lane number and adjust error data: (1) missing data between first and second rows, e.g., “20130201154130” in the lane 1; (2) speed value abruptly changed at timestamp “20130201154200” in lane 2; and (3) negative number at timestamp “20130201154130” in lane 3. These noise data are replaced with the nearest neighbors as depicted in Figure 1b. For the resampling, the filtered speed values in separate lanes are combined using Equation (1) and the number of passing cars is calculated using Equation (2). Finally, the four sensor values per 30 s interval are resampled into two sensor values using Equation (3) (Figure 1c).

## 3. Traffic Flow Modeling

We developed a statistical model that can precisely predict the traffic flow at *t + k* timestamp, which is established through analyzing the historical data and geometric correlation of traffic flow. In this paper, a *3D heat map is defined that describes the traffic conditions between roads*. This 3D heat map can effectively represent the correlations between spatially- and temporally-adjacent traffic states. Here, only the vehicle speed is considered in the traffic flow prediction.

Let *S* be the 3D volume of $M_{1}\times M_{2}$ irregular lattices, which is represented as $S=\left\{S_{st}\right\}$. An element $S_{st}$ indexes a cone zone at site *s* and time *t*. Then, the heat map (*H*) is defined for the *S*, and its element represents the traffic conditions, e.g., speed or congestion level. Let Λ be the set of traffic conditions, where a smaller value indicates a lower speed and more traffic congestion. 

That is, the heat map is written as follows:

$$H=\{H_{st}|H_{st}\in \Lambda \;and\;1\leq s\leq M_{1}\times M_{2}\},\;\mathrm{where}\;\Lambda =\left\{\lambda_{1},\lambda_{1},\cdots ,\lambda_{R}\right\}$$

In KEC, the traffic condition is categorized into three levels of free, slow, and congested, *i.e.*, *R =* 3. In this study, we split Λ into twelve levels in order to represent the detailed traffic conditions. It is also assumed that the speed range is from 1 to 120 km/h (*i.e.*, the speed limit). We calculate the traffic condition using the equation $\left\lfloor V_{m}/10-1\right\rfloor$. For example, if the current speed is 100 km/h, the traffic condition is 9. 

Let $\Gamma =\left\{\eta_{st}\right\}$ be a spatio-temporal neighborhood in *S*, where $\eta_{st}$ is the set of neighboring sites *(s,t)*. X is the traffic condition in *S*. an element of *X*, *i.e.*, $X_{st}$ takes a value from a different set Λ. In general, the traffic flow of one road is affected by the traffic conditions of the previous time stamp, and it is also affected by the traffic conditions of the geometrically-connected roads [6,7,12,13]. Thus, the 3D heat map is modeled using a spatio-temporal Markov random field (spatio-temporal MRF, 3D MRF) [27,28], because it satisfies the following Markovian property:

$$\left(H_{st}=h_{st}|H_{qr}=x_{qr},\left(s,t\right)\neq \left(q,r\right)\right)=P\left(H_{st}=h_{st}|H_{qr}=x_{qr},\left(q,r\right)\in \eta_{st}\right)$$

**Figure 2:**
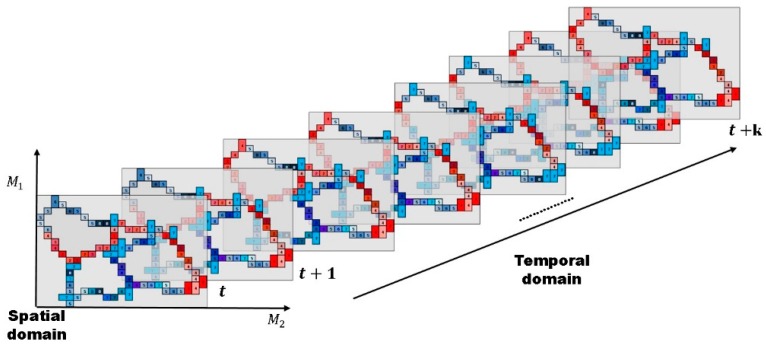
Definition of the 3D heat map: the heat map is defined on the irregular lattice and represents the spatial and temporal correlation of the traffic flow. A cell on the map corresponds to each road (or link, cone-zone), and a different color is assigned to each cell according to its traffic conditions (e.g., speed and density). As can be seen, the cells have similar colors to their spatial and temporal neighbors. Based on these characteristics, we model the traffic flows as 3D MRFs and then estimate the traffic flow at time *t + k*.

Figure 2 presents an example of a 3D heat map. As seen in the figure, the heat map is defined on the irregular lattice, and its basic element, *i.e.*, a cone zone, has different colors according to its traffic conditions. Then, observe that the spatially- and temporally-adjacent cells have the same or similar colors. This represents the spatio-temporal correlation between adjacent cone zones.

**Figure 3:**
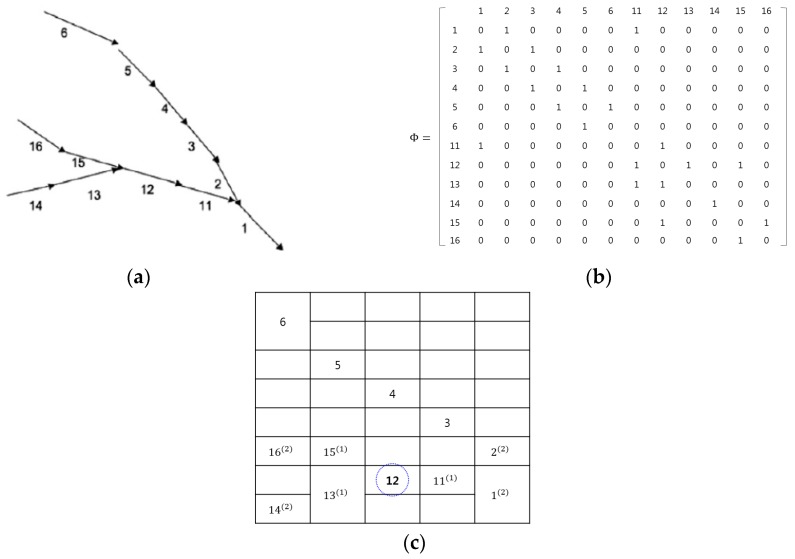
Example of map representations: (**a**) real map; (**b**) Wynter *et al.*’s adjacency matrix; and (**c**) proposed heat map.

Figure 3 illustrates the heat map generated from road environments, where the map was obtained using Wynter *et al.*’s work [6]. The common aspect of the two works is the consideration of the spatial interactions between roads. From Figure 3a, the adjacency matrix was drawn and is depicted in Figure 3b. In the adjacency matrix, the spatial interactions between roads are only considered for the roads that are directly connected to each other. For example, the element (i,j) has a non-zero value if road *i* is a neighbor of road *j*. In contrast, the heat map in Figure 3c is constructed using our method. The proposed method can represent all possible interactions among roads through using the high-order neighborhood system, as well as the relationships between the directly connected roads.

Let $\eta_{ij}$ be the neighbors of pixel (*i*, *j*) in the spatial domain. Then, for the heat map (*H*) on the lattices $M_{1}\times M_{2}$, the neighbor set of (*i*, *j*) is defined as the set of nearby cone zones within a diameter of *d*.

$$\eta_{ij}=\left\{\left(k,\;l\right)\in H|0\leq {\left(k-i\right)}^{2}+{\left(l-j\right)}^{2}\leq d\right\}$$

For example, see road ‘12’ in Figure 3c: using the first-order neighborhood system, it has three neighbors of $\left\{11^{1},\;13^{1},\;15^{1}\right\}$, which are the same neighbors as in Wynter *et al.*’s study [6]. Furthermore, for the second-order neighborhood system, it has seven neighbors of $\left\{11^{2},\;13^{2},\;15^{2},\;1^{2},\;2^{2},\;14^{2},\;16^{2}\right\}$. Therefore, in the proposed method, the spatial interactions among the roads can be expanded using the high-order neighbor systems, which is explained in Section 4.1 (cliques), and then different weights are assigned to every spatial interaction using example-based learning (which is explained in Section 4.2).

## 4. Parameter Estimation and Prediction

Our goal is to predict the $H_{t+k}$ given the temporal and spatial domain parameters; that is:

(4) $$H_{t+k}=\left\{h_{t+k}\left(r\right)|r\in cone-zone\right\},\;\text{where}\;h_{t+k}\left(r\right)\in \Lambda$$

where $H_{t+k}$ is the 3D heat map at time *t* consisting of *r* cone zones. $H_{t+k}\left(r\right)$ is a mapping function from the traffic historical data to the predicted traffic condition.

As illustrated in Equation (4), the heat map is modeled using the 3D MRF. Thus, the Markov process is used for the traffic flow estimation. According to Hammersley–Clifford’s theorem [27,28], the probability $P(h_{t+k}|h_{t},A)$ has the following Gibbs distribution:

(5) $$P\left(h_{t+k}|h_{t},A\right)=\frac{1}{z}exp\left\{-E\left(h_{t+k}|h_{t},A\right)\right\}$$

where $E(h_{t+k}|h_{t},A)$ is the energy function for predicting $h_{t+k}$ given the model parameter *A* and historical data $h_{t}$. Because the multilevel logistic model is used, the energy function is defined as the sum of the spatial clique potentials, $S_{c}\left(h_{t+k}|h_{t},A\right)$, and the temporal clique potentials, $T_{c}\left(h_{t+k}|h_{t},A\right)$; thus, Equation (5) is rewritten as follows:

(6) $$E\left(h_{t+k}|h_{t},A\right)=\underset{c\in C}{\sum }\left\{S_{c}\left(h_{t+k}|h_{t},A\right)+T_{c}\left(h_{t+k}|h_{t},A\right)\right\}$$

where the *C* is the cliques defined in the spatio-temporal neighborhood Γ. 

As depicted in Equation (6), the energy function is obtained from the summation of two potentials over all possible cliques: spatial potentials $S_{c}\left(h_{t+k}|h_{t},A\right)$ and temporal potentials $T_{c}\left(h_{t+k}|h_{t},A\right)$. The former imposes the spatial continuity of the traffic condition on the neighbors; the latter achieves the temporal continuity of the traffic condition.

### 4.1. Cliques

**Figure 4:**
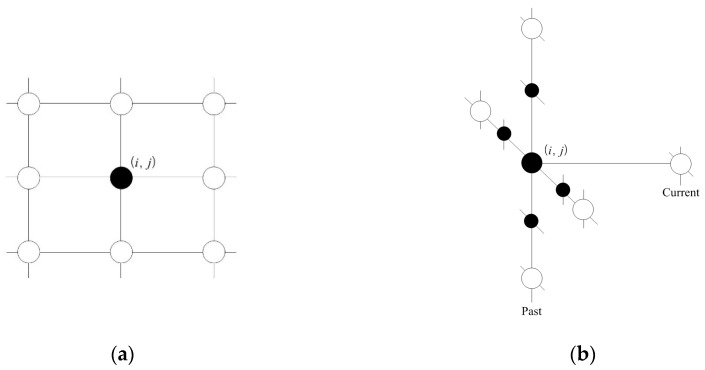
Second–order spatio-temporal neighborhood system: (**a**) spatial domain and (**b**) temporal domain.

A clique (*c*) is defined as a set of cone zones in which all pairs are mutual neighbors. In Equation (6), *C* is a possible set of cliques on spatio-temporal domain. Figure 4 illustrates the second-order spatio-temporal neighborhood system. In this study, the proposed model assumes that only the non-zero potentials are those that correspond to the two-pair cliques and triple cliques.

**Figure 5:**
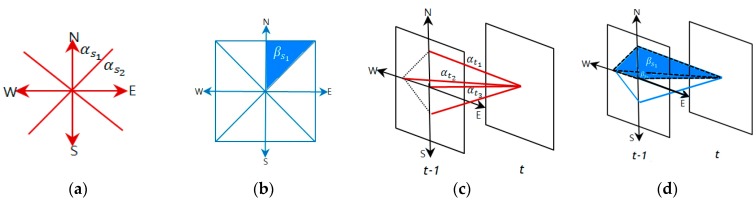
Cliques identified in the spatio-temporal domain. (**a**,**b**) are two-pair cliques and triple cliques in the spatial domain; (**c**,**d**) are two-pair and triple cliques in the temporal domain. The cliques represent the temporal or spatial dependencies on the traffic conditions.

Figure 5 presents the cliques defined in the spatio-temporal domain using the proposed method. As seen in Figure 5a,b, the two-pair and triple cliques in the spatial domain are identified to represent the correlation between the cone zone and its second-order neighbors at the same timestamp, respectively. Figure 5c,d depict the two-pair and triple cliques defined in the temporal domain using the first-order neighborhood system. These cliques indicate the relationships between the cone zone at time *t + k* and its neighborhoods at time *t*. 

### 4.2. Model Parameter Estimation

The parameter set A={α, β} represents the weights that indicate the size of the effects on the next traffic prediction. In parameter set *A*, α is the parameter for two-pair cliques and β is the parameter for triple cliques. Furthermore, the parameter subscripts indicate the defined domain, e.g., subscript *s* and *t* indicate the spatial domain and the temporal domain, respectively. In the parameter set, larger weights signify greater influence on the prediction. These model parameters were estimated using example-based learning such as regression [17]. 

Figure 5 presents the cliques used in this study. As seen in the figure, these cliques represent the temporal and spatial dependency on the traffic conditions. Then, the parameters are assigned to the respective cliques. Based on these cliques, the spatial and temporal potentials are defined as in Equations (7) and (8), respectively:

(7) $$\begin{array}{cc} T_{c}\left(h_{t+k}|h_{t},A\right) & =\alpha_{T}P_{T}\left(h\left(r,t\right),h\left(r,t+k\right)\right)+\alpha_{T}\underset{q_{1}\in \eta_{s}\left(r\right)}{\sum }P_{T}\left(h\left(r,t+k\right),h\left(q_{1},t\right)\right)\\ & +\beta_{T}\underset{\left(q_{1},q_{2}\right)\in \eta_{t}\left(r\right)\&q_{1}\neq q_{2}}{\sum }P_{T}\left(h\left(r,t\right),h\left(q_{1},t+k\right),h\left(q_{2},t+k\right)\right) \end{array}$$

(8) $$\begin{array}{cc} S_{c}\left(h_{t+k}|h_{t},A\right) & =\alpha_{S}\underset{q_{1}\in \eta_{s}\left(r\right)}{\sum }P_{S}\left(h\left(r,t\right),h\left(q_{1},t\right)\right)\\ & +\beta_{S}\underset{\left(q_{1},q_{2}\right)\in \eta_{s}\left(r\right)\&q_{1}\neq q_{2}}{\sum }P_{S}\left(h\left(r,t\right),h\left(q_{1},t\right),h\left(q_{2},t\right)\right) \end{array}$$

where $P_{T}\left(\cdot \right)$ and $P_{S}\left(\cdot \right)$ denote the potential functions for the cliques in the temporal and spatial domains, respectively.

In order to select the clique potential function $P_{T}\left(\cdot \right)$ and $P_{S}\left(\cdot \right)$, three functions are considered:

- P(x, K)=log(cosh(dist(x, K)),
- $P\left(x,\;K\right)=log(1+{\left(dist\left(x,\;K\right)\right)}^{2})$*, and*
- $P\left(x,K\right)=min\left(\left|{\left(dist\left(x,K\right)\right)}^{2}\right|,\left|2\left(dist\left(x,\;K\right)\right)-1\right|\right)$

where $dist\left(x,\;K\right)=\frac{1}{m}\sum_{i=1}^{m}{\left(x-k_{i}\right)}^{2}$ is a function of the average Euclidean distance between the traffic conditions at the cone zones in the clique; *x* is the traffic condition in the current cone zone; and K is the set of traffic conditions in the neighborhood of cone zone *x* on the spatio-temporal domain, e.g., $K=\left\{k_{1},\cdots \;k_{m}\right\}$. The size of K, *i.e.*, *m*, is the size of the neighbor set. For example, when *m* = 1, it is a two-pair clique such as $C_{2}=\left\{x,\;k_{1}\right\}$. 

The experimental results demonstrated that the function of $\log(1+{\left(dist\left(x,\;k_{1},\cdots \;k_{m}\right)\right)}^{2})$ performed best. Thus, this function was adopted for the remainder of the study.

Now, an example for the energy function is provided. In Figure 3c, see road “12”; the two-pair cliques and triple cliques in the spatio-temporal domain can be represented as in Equations (9) and (10), respectively:

(9) $${C_{s}}^{\left(2\right)}=\{\alpha_{S_{1}}\left\{h\left(11,\;t\right),h\left(12,\;t\right)\right\},\;\alpha_{S_{2}}\left\{h\left(12,\;t\right),\;h\left(13,\;t\right)\right\},\alpha_{S_{3}}\left\{h\left(12,\;t\right),\;h\left(15,\;t\right)\right\}\},\;{C_{s}}^{\left(3\right)}=\{\beta_{S_{1}}\left\{h\left(1,\;t\right),\;h\left(11,\;t\right),\;h\left(12,\;t\right)\right\},\beta_{S_{2}}\{h\left(2,\;t\right),\;h\left(11,\;t\right),\;h\left(12,\;t\right)\},\;\beta_{S_{3}}\left\{h\left(12,\;t\right),\;h\left(13,\;t\right),\;h\left(15,\;t\right)\right\},\;\beta_{S_{4}}\left\{h\left(12,\;t\right),\;h\left(15,\;t\right),\;h\left(16,\;t\right)\right\},\beta_{S_{5}}\left\{h\left(12,\;t\right),\;h\left(13,\;t\right),\;h\left(14,\;t\right)\right\}\}$$

(10) $${C_{T}}^{\left(2\right)}=\{\alpha_{T_{1}}\left\{h\left(12,\;t\right),\;h\left(12,\;t+k\right)\right\},\alpha_{T_{2}}\left\{h\left(11,\;t\right),\;h\left(12,\;t+k\right)\right\},\;\alpha_{T_{3}}\left\{h\left(13,\;t\right),\;h\left(12,\;t+k\right)\right\},\alpha_{T_{4}}\left\{h\left(15,\;t\right),\;h\left(12,\;t+k\right)\right\}\},\;{C_{s}}^{\left(3\right)}=\left\{\beta_{T_{1}}\left\{h\left(12,\;t\right),\;h\left(12,\;t+k\right),\;h\left(11,\;t\right)\right\},\;\beta_{T_{2}}\left\{h\left(12,\;t\right),\;h\left(12,\;t+k\right),\;h\left(13,\;t\right)\right\},\beta_{T_{3}}\left\{h\left(12,\;t\right),\;h\left(12,\;t+k\right),\;h\left(15,\;t\right)\right\}\right\}$$

As seen in Figure 5 and Equations (9) and (10), the proposed method allocates different weights according to each clique defined in the spatio-temporal interaction, which is called a “clique parameter”. Then, the potential function in Equation (11) can be rewritten as the sum of the clique potential for all cliques. We assumed that the current cone zone is 12, *i.e.*, h(12, t+k). Therefore, we should estimate 4 $\alpha_{T}$s and 3 $\alpha_{S}$s for the two-pair cliques and 3 $\beta_{T}$s and 5 $\beta_{S}$ for the triple cliques. Thus, Equation (3) can be written as follows:

(11) $$\begin{array}{lll} S_{c}\left(h_{t+k}|h_{t},Α\right) & = & \alpha_{S_{1}}\cdot \log\left(1+dist{\left(h\left(11,\;t\right),h\left(12,\;t\right)\right)}^{2}\right)+\;\alpha_{S_{2}}\\ & & \cdot \log\left(1+dist{\left(h\left(12,\;t\right),\;h\left(13,\;t\right)\right)}^{2}\right)+\alpha_{S_{3}}\\ & & \cdot \log\left(1+dist{\left(h\left(12,\;t\right),\;h\left(15,\;t\right)\right)}^{2}\right)+\beta_{S_{1}}\\ & & \cdot \log\left(1+dist{\left(h\left(1,\;t\right),\;h\left(11,\;t\right),\;h\left(12,\;t\right)\right)}^{2}\right)+\;\beta_{S_{2}}\\ & & \cdot \log\left(1+dist{\left(h\left(2,\;t\right),\;h\left(11,\;t\right),\;h\left(12,\;t\right)\right)}^{2}\right)+\beta_{S_{3}}\\ & & \cdot \log\left(1+dist{\left(h\left(12,\;t\right),\;h\left(13,\;t\right),\;h\left(15,\;t\right)\right)}^{2}\right)+\beta_{S_{4}}\\ & & \cdot \log\left(1+dist{\left(h\left(12,\;t\right),\;h\left(15,\;t\right),\;h\left(16,\;t\right)\right)}^{2}\right)+\beta_{S_{5}}\\ & & \cdot \log\left(1+dist{\left(h\left(12,\;t\right),\;h\left(13,\;t\right),\;h\left(14,\;t\right)\right)}^{2}\right) \end{array}$$

(12) $$\begin{array}{lll} T_{c}\left(h_{t+k}|h_{t},A\right) & = & \alpha_{T_{1}}\cdot \log\left(1+dist{\left(h\left(12,\;t\right),\;h\left(12,\;t+k\right)\right)}^{2}\right)+\alpha_{T_{2}}\\ & & \cdot \log\left(1+dist{\left(h\left(11,\;t\right),\;h\left(12,\;t+k\right)\right)}^{2}\right)+\alpha_{T_{3}}\\ & & \cdot \log\left(1+dist{\left(h\left(13,\;t\right),\;h\left(12,\;t+k\right)\right)}^{2}\right)+\alpha_{T_{4}}\\ & & \cdot \log\left(1+dist{\left(h\left(15,\;t\right),\;h\left(12,\;t+k\right)\right)}^{2}\right)+\beta_{T_{1}}\\ & & \cdot \log\left(1+dist{\left(h\left(12,\;t\right),\;h\left(12,\;t+k\right),\;h\left(11,\;t\right)\right)}^{2}\right)+\beta_{T_{2}}\\ & & \cdot \log\left(1+dist{\left(h\left(12,\;t\right),\;h\left(12,\;t+k\right),\;h\left(13,\;t\right)\right)}^{2}\right)+\beta_{T_{3}}\\ & & \cdot \log\left(1+dist{\left(h\left(12,\;t\right),\;h\left(12,\;t+k\right),\;h\left(15,\;t\right)\right)}^{2}\right) \end{array}$$

where the range of these clique parameters is $0\leq \alpha_{S},\alpha_{T},\beta_{S},\beta_{T}\leq 1$.

Finally, the energy function is calculated using the sum of the spatial and temporal potentials as depicted in Equations (11) and (12).

## 5. Experiments

In order to evaluate the performance of the proposed system, we collected traffic data from the Korean expressway corporation. The collected data were processed in order to remove noise and reduce errors. Then, the traffic flow prediction was performed.

### 5.1. Data Collection

**Figure 6:**
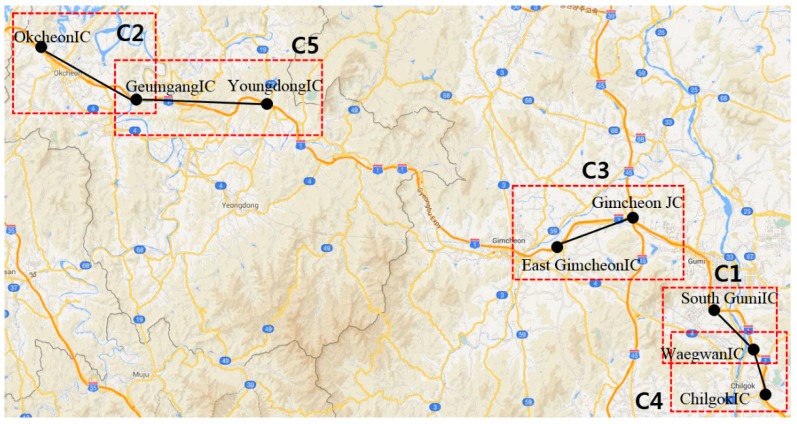
Korean expressways.

The experimental data were provided by the Korean Expressway Corporation (KEC), which manages the traffic conditions of Korean expressways and analyzes the data. The KEC collects a large volume of traffic flow from ten expressways. Figure 6 illustrates the ten Korean expressways, which are denoted by the orange lines. Among the ten expressways, the proposed method was first applied to the Gyeongbu expressway, which is the backbone of the Korean traffic flow and has the largest daily traffic volume. The Gyeongbu expressway consists of 1076 cone zones. Due to the space constraints, this paper reports the prediction results for five cone zones, which are marked by red rectangles in Figure 6.

For the model training and testing, we collected the traffic data from February to May 2013. In order to reflect the seasonal trends, the traffic prediction model is updated at one-month intervals. Thus, the traffic data collected from the first half of one month were used for model training, and the other data were used for testing the performance of the prediction model.

Table 1 presents the identification data of the five cone zones used in this experiment. According to the traffic volume, KEC installed different numbers of lanes in the cone zones. As mentioned in Section 2, the traffic data was collected in units of lanes in the respective cone zones. Each VDS collects 2880 samples per day. Thus, the traffic data were re-calculated through data preparation and filtering. Then, in order to reduce the prediction error caused by the noise and missing data, we used the nearest-neighbor interpolation and statistical process.

**Table 1:** Details of the five cone zones used in these experiments.

	Cone-Zone ID	Cone-Zone Name	# of Lanes
*C1*	0010VDS10100	South GumiIC–WaegwanIC	4
*C2*	0010VDS18800	OkcheonIC–GeumgangIC	3
*C3*	0010VDS13100	East GimcheonIC–GimcheonJC	3
*C4*	0010VDS09800	WaegwanIC–ChilgokIC	4
*C5*	0010VDS17800	GeumgangIC–YoungdongIC	3

In this paper, the traffic flow is calculated by harmonic average using average speed and number of vehicles at time t and then quantized into 12 levels. It means that a continuous random variable is converted into the discrete random variable. To find the proper stochastic process to our data, we extracted resampled data and performed Chi-square goodness of fit test.

**Figure 7:**
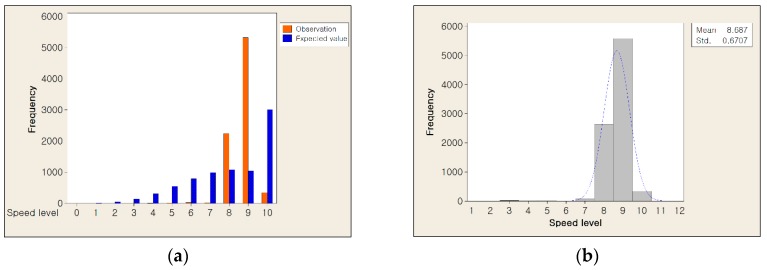
The analysis results of traffic flow data (**a**) Chi-square goodness of fit test result and (**b**) the distribution of observed traffic flow data mapped with Gaussian distribution.

Figure 7 shows the analysis results with Poisson distribution and Gaussian distribution. As shown in Figure 7a, the distribution of the observed data was not matched with the distribution of expected data using Poisson distribution. As illustrated in Figure 7b, the observed data had Gaussian distribution whose mean is about 8.7 and the standard deviation is about 0.7, respectively. Therefore, we assume that our data is fit to a Gaussian distribution.

**Figure 8:**
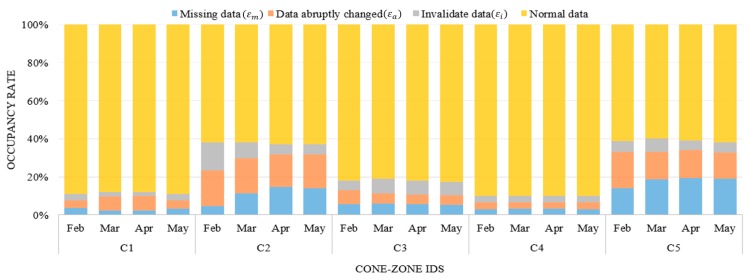
Data distribution of noise during four months at the respective cone zones.

Figure 8 illustrates the data distribution during the four investigated months for the respective cone zones, in which the data was classified as normal data or noise data. As illustrated in Section 2, the data can contain three types of the noise: missing data ($\varepsilon_{m}$), abruptly changed data ($\varepsilon_{a}$), and invalidated data ($\varepsilon_{i}$). Then, the rate of total noise occurrences is calculated using following equation:

$$\text{Rate of total noise occurrences }=\frac{\left(\varepsilon_{m}+\varepsilon_{a}+\varepsilon_{i}\right)}{N}$$

where *N* is the total number of sensor data collected during one month. In most cases, the errors primarily resulted from abruptly changing vehicle speeds. After the error correction, the data obtained from cone zones “*C2*” and “*C5*” were two-fold larger than the other three cone zones. Moreover, the two cone zones “*C2*” and “*C5*” had errors due to missing data, which increased the difficulty of predicting the next traffic flow. More details are provided in Section 5.3. The filtered data are combined using the harmonic average, and then the sensor values are aggregated into 1 min average values. As a result, 1440 samples were recorded to the database daily for each cone zone.

### 5.2. Model Parameter Estimation

Given the traffic data in the respective cone zones, the heat map should be first constructed based on the geometric information. 

**Figure 9:**
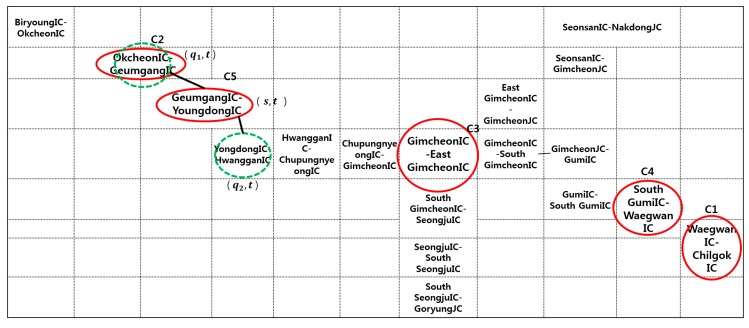
Heat map defined based on the selected five cone zones.

Figure 9 presents the generated heat map from Biryoung IC to Chilgok IC on the Gyeongbu expressway. As depicted in Figure 8, each cell corresponds to a cone zone. The different sizes are assigned according to the length of the cone zone. The red circles in the heat map indicate the five selected cone zones, which were defined in Table 1. Then, for example, if the traffic condition in cone zone “*C5*” (s, t) is predicted, the proposed method uses the two neighborhoods “OkcheonIC-GeumgangIC” $\left(q_{1},\;t\right)$ and “GeumgangIC-YoundongIC” $\left(q_{2},\;t\right)$ in the spatial domain, which are denoted by the green circles in Figure 9.

From the heat map, a graph can be constructed for the cone zones, where the nodes correspond to the cone zones and the edge indicates the geometrical relationships between the cone zones. Then, the two-pair cliques and triple cliques in the spatio-temporal domain are defined using the *n*th-order neighborhood system.

**Figure 10:**
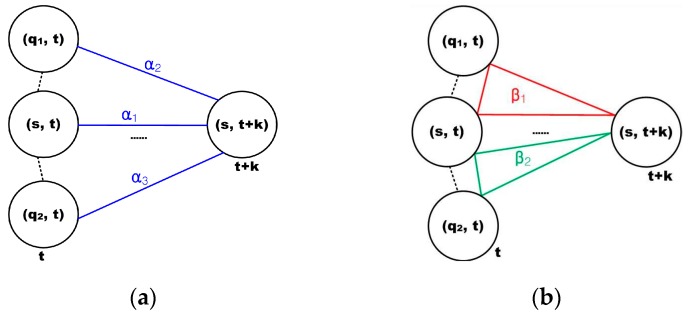
Model parameters at each cone zone: (**a**) description of the clique parameters at two-pair cliques and (**b**) illustration of the clique parameters for the triplet cliques.

Once the heat map is constructed, the traffic flow prediction in a cone zone is formulated as the minimization of the energy function of Equation (6). In order to calculate the energy function, the model parameter A should be estimated. As illustrated in Section 4, only the two-pair and triple cliques are considered in the spatial and temporal domains, respectively. Thus, five model parameters are required in order to estimate the traffic flow at each cone zone, and these are described in Figure 10.

**Figure 11:**
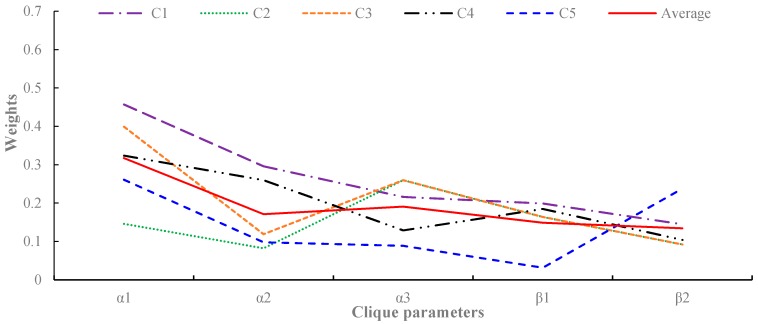
Estimations of the model parameters for the cliques in Figure 9.

Figure 11 presents examples of the model parameter estimation results. On average, the five parameters were set to 0.32, 0.17, 0.19, 0.15, and 0.13. From Figure 11, some interesting points can be observed. First, most cone zones have the largest correlation in parameter $\alpha_{1}$, which is a dependency factor between the same locations at different timestamps. This indicates that the temporal correlation has a more significant influence on predicting the traffic flow than the other correlations. Second, although $\alpha_{1}$ had relatively larger values than the other parameters, the weights between the spatially-adjacent cone zones, such as $\alpha_{2}$ and $\alpha_{3}$, have values of 0.1 to 0.3, respectively. This indicates that both the spatial and temporal correlations should be considered in order to accurately predict traffic flows. Third, in addition, the cone zones have similar trends in the parameter distributions. The average distribution is marked with a red line in Figure 10. When compared with the average distribution, most cone zones have a similar distribution to the average line. However, C2 and C5 have different trends in the parameter distribution, which was potentially caused by noisy data. Excluding C2 and C5, the variation of the five parameters between cone zones were 0.15, 0.31, 0.26, 0.09, and 0.2.

### 5.3. Prediction Results

In the proposed method, the traffic flow prediction is formulated as the minimization of the energy function of Equation (6). In order to quantitatively evaluate the performance of the proposed method, the prediction results were compared with the real traffic data.

**Figure 12:**
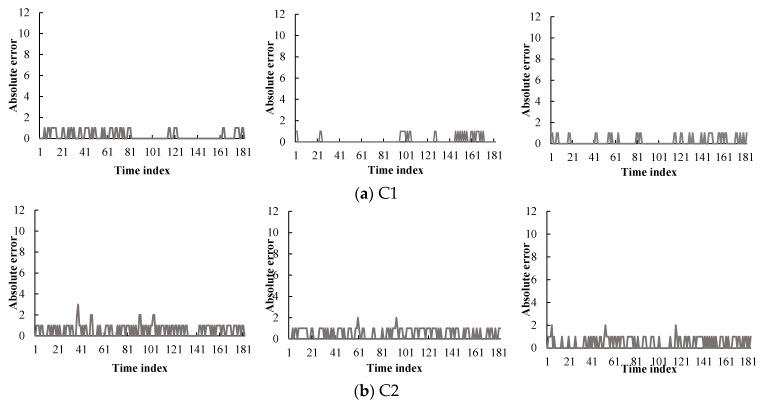

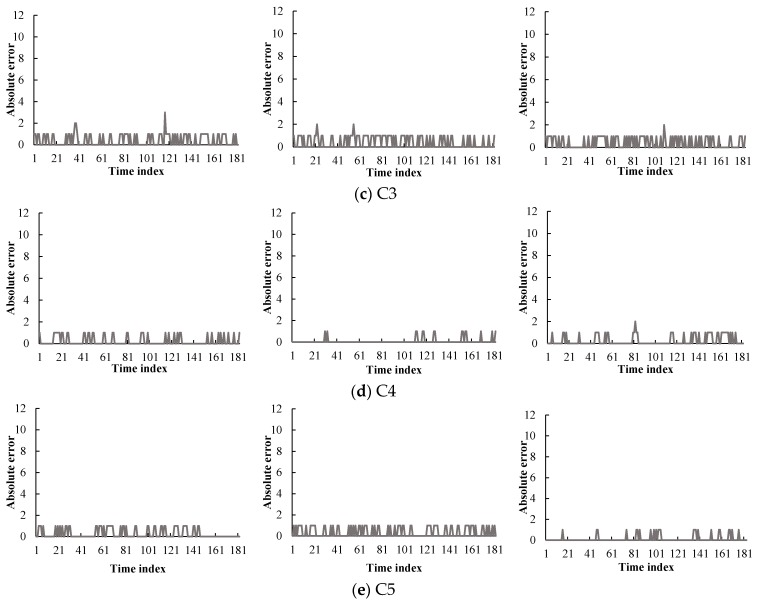
Traffic flow prediction errors at five cone-zones during February (**left**), March (**middle**), and April (**right**), respectively.

Figure 12 presents a time series plot of the traffic condition prediction errors against the actual traffic conditions from February to April 2013. The x-axis is the time index that was chosen: from 6 P.M. to 9 P.M., one weekday. This period has 181 data points and it covered the evening peak hours of traffic. The y-axis is the absolute difference between the actual traffic condition and the predicted condition, which consists of twelve levels. As depicted in Figure 12, the errors are denoted by a solid line. Most graphs demonstrate that the predicted values match the real data well. In contrast, many errors were generated at the cone zones *C2* and *C5* (see Figure 12b,e, respectively). These errors appear to be caused by noisy data due to missing data and abruptly changing vehicle speeds. As depicted in Figure 8, these cone zones exhibited higher occupancy rates of the noise than other cone zones. In order to manage this problem, we are developing an efficient noise filtering method as part of our ongoing current research.

Table 2 presents the overall accuracy of the respective cone zones in different months. The average accuracy was approximately 85%. In particular, in the case of low noise occupancy, the proposed method exhibited an accuracy of above 90%. This proves the effectiveness of the proposed method.

**Table 2:** Accuracy of the traffic flow prediction (%).

Cone-Zone ID	February	March	April	May	Average
*C1*	93	99.9	92.8	93.1	94.7
*C2*	65.5	85.1	74	72.9	74.4
*C3*	78.3	88.2	75.9	82.7	81.3
*C4*	91.3	94.8	89.1	88.9	91
*C5*	82.9	81.4	81.3	79.9	81.4

### 5.4. Performance Comparison

In this experiments, three other methods were adopted as baselines: Yundin *et al.*’s method (Baseline 1, 2009) [24], Gong *et al.*’s method (Baseline 2, 2002) [15], and Liebig *et al.*’s method (Baseline 3, 2012) [23]. 

Baseline 1 used only the time series data to estimate the traffic flow, where MRF models is used to characterize the dependency between traffic flows of the one road at the different time steps. In this method, the parameter α determines the impact of the historical traffic flows to the current states, which was tuned within 0–1 and was set to 0.5 by experiments. Baseline 2 is one of the most famous non-parametric methods, where *K*-NN is used to predict the traffic flow at the next time step. Then, the neighbors are defined as the cone zones that satisfy the following conditions: first, the cone zones should be adjacent on the spatial-temporal domain; second, the cone zones have the high correlations with the target cone zone. To choose the optimal *k* for *K*-NN, we performed the test while tuning the *k* from 1 to 10. Then, the result is converged when the *k* is set to above seven. The larger *k* requires more computational time for comparison, thus we set the value to seven. Baseline 3 employed Gaussian process regression, which has been a popular method in traffic volume estimation and can estimate the traffic flow as the quantities on the roads. In that method, the combinatorial Laplacian matrix *L* represents the relationship between the target cone zone and the adjacent cone zones in the spatial-temporal domain, where the dimension of the matrix *L* determines how many adjacent neighbors are necessary to predict the traffic flow at next time steps. To find the optimal dimension d of the matrix *L*, we performed the test while tuning the d within 1 to 10. Then the parameter is set to six by experiments. 

Baseline 1 was designed to compare the proposed spatiotemporal method with the time series method. In contrast, Baseline 2 and Baseline 3 uses both geometric and temporal correlations of adjacent roads; however, they used the different mechanisms for traffic flow estimation to the proposed method: Baseline 2 used *K*-NN and Baseline 3 used for Gaussian process regression, respectively. Thus, through the performance comparison among the three methods, we can demonstrate the effectiveness of the proposed prediction models based on the probabilistic model such as spatiotemporal MRF. 

Using four methods, the experiments for predicting the numerical data such as vehicle speed or number of cars on the roads were performed. In experiments, we first converted the numerical data to the categorical data for the comparison with the proposed method. Then, the existing method performed traffic flow prediction using the same data. To compare the prediction errors of the proposed method, we measured the residual errors under four different methods. 

**Figure 13:**
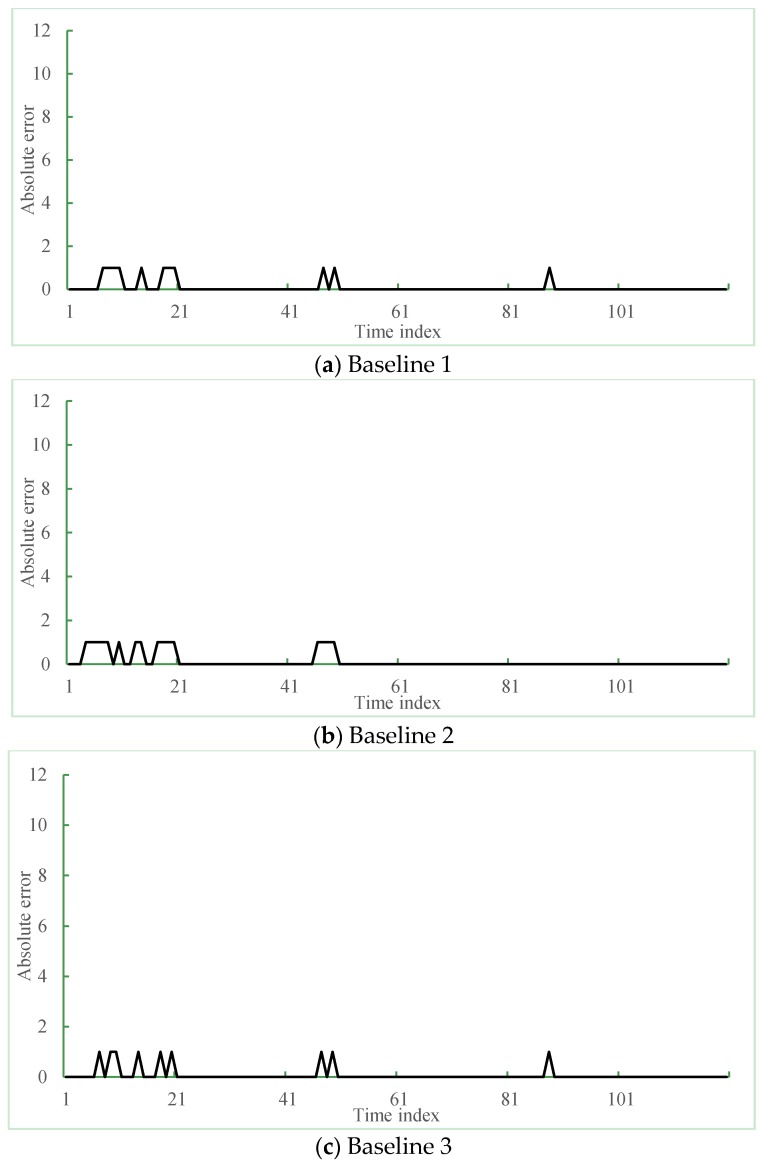

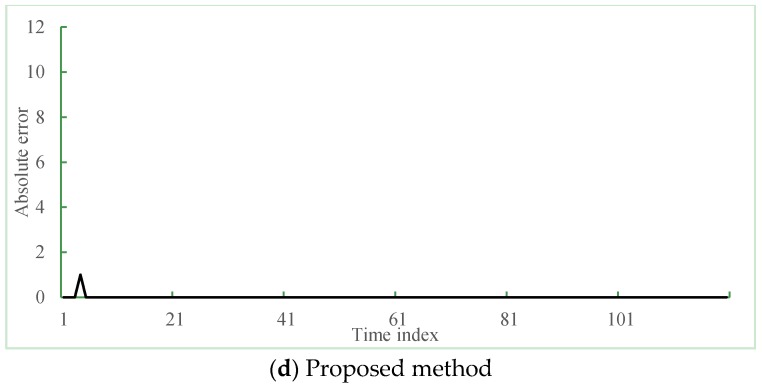
Performance comparison between three existing methods and the proposed method: (**a**–**c**) prediction errors of three baseline methods and (**d**) the proposed method.

Figure 13 shows the prediction residual of the proposed and the three different methods. In Figure 13, the x-axis is the time index that was selected: 120 min from 6 P.M. to 8 P.M. on a weekday and the y-axis is the absolute error between ground truth and the predicted result, respectively. Figure 13a–d present the residual error results of the *K*-NN based method, time series-based method, Gaussian process regression-based method and the proposed method, respectively. The peaks on the graph mean the occurred errors of the prediction results. As shown in Figure 13a,c, the average prediction errors of three methods occurred about 12 times during two hours. However, the proposed method showed accurate prediction results.

**Table 3:** Statistical comparison between the proposed method and three baselines.

	Methods	Total	Baseline 1		Baseline 2		Baseline 3		Proposed Method	
Months			# of Hits (%)	# of Errors (%)	# of Hits (%)	# of Errors (%)	# of Hits (%)	# of Errors (%)	# of Hits (%)	# of Errors (%)
February		20,160	11,189 (55.5%)	8972 (44.5%)	11,836 (58.7%)	8324 (41.3%)	16,301 (80.9%)	3859 (19.1%)	19,092 (94.7%)	1069 (5.3%)
March		22,320	12,522 (56.1%)	9799 (43.9%)	13,018 (58.3%)	9302 (41.7%)	20,648 (92.5%)	**1672 (7.5%)**	16,606 (74.4%)	**5714 (25.6%)**
April		21,600	12,183 (56.4%)	9418 (43.6%)	10,656 (49.3%)	10,944 (50.7%)	13,832 (64%)	7768 (36%)	17,561 (81.3%)	4039 (18.7%)
May		22,320	12,397 (55.5%)	9924 (44.5%)	10,397 (46.6%)	11,923 (53.4%)	14,124 (63.3%)	8196 (36.7%)	20,311 (91%)	2009 (9%)
Average		21,600	12,073 (55.9%)	9528 (44.1%)	11,499 (53.2%)	10,101 (46.8%)	16,237 (75.2%)	5363 (24.8%)	18,393 (85.2%)	3207 (14.8%)

Table 3 summarizes the statistical comparison of the prediction results between the abovementioned three existing methods and the proposed method. We used half-month data as test data collected from the cone zones. In the Table 3, the number of hits indicates the count of exact matches between ground truth and the predicted results and the number of errors is measured by counting the mismatches between them, respectively. When compared with Baseline 1 and Baseline 2, the proposed method showed considerable decreased number of errors *i.e.*, about one-third of errors. On average, the Baseline 2 were the lowest accuracy of 53% among four methods. The Baseline 1 and the Baseline 3 showed the accuracies of 56% and 75%, respectively. The proposed method showed the highest accuracy of 85.1% and achieved a performance improvement of 113% compared with the Baseline 3. Thus, the experimental results confirmed the effectiveness of the proposed method. 

In addition, to be useful for the traffic flow estimation system, the proposed method can be operable in real-time running of the model. Thus, the computation time of the model is a critical consideration. Table 4 below shows comparison of computation time using three existing methods and the proposed method for the model parameter estimation and the prediction. The computation times were measured for estimating the model parameter and predicting the traffic flow during a half month using a desktop (3.0 GHZ CPU and 4 GB RAM). In case of Baseline 2, the model parameter estimation were performed to find the correlative cone zones among the neighbors on the spatiotemporal domain. The calculation time for estimating the model parameters using Baseline 3 took the longest, among the four methods. The calculation time for the traffic flow prediction using the proposed method took about 0.01. Since Baseline 2 measures Euclidian distances between the correlative cone zones, it requires the longest time for the traffic flow prediction. For all cone-zones on the Gyeongbu expressway, the calculation time for the prediction using the proposed system takes about 10 s and is smaller than the interval of the VDS sensor (30 s).

**Table 4:** Comparison of computational time using four methods at one cone-zone on the Gyeongbu expressway.

Methods	Model Parameter Estimation	Single Run in Real-Time Using Calibrated Model, on One Cone Zone (in Second)
Baseline 1	***25***	***0.01***
Baseline 2	***60***	***0.1***
Baseline 3	***2800***	***0.07***
Proposed method	***30***	***0.01***

Consequently, the comparison results confirmed the efficiency and effectiveness of the proposed method. In addition, the results showed that the proposed method was practical for Intelligent Traffic System.

## 6. Conclusions

Traffic flow prediction that provides short- and long-term forecasts using real-time data is an essential component in controlling traffic in ITSs. In this paper, a statistical method that predicts traffic flows was proposed. For more accurate prediction, the proposed method used the correlation from both the temporal and spatial domains. The proposed method is composed of three modules: data preparation and filtering, 3D heat map modeling, and parameter estimation and prediction. The traffic data collected from several VDSs include noise and errors; thus, we first performed noise filtering and resampling using interpolation and statistics. Then, the heat map was constructed from the expressways and modeled using a 3D Markov random field (MRF). Based on the Markov process, the traffic flow was predicted. In this stage, the spatial and temporal correlation parameters between adjacent roads were first calculated using example-based learning. Using these model parameters, the prediction was performed through minimizing the energy function. In order to assess the validity of the proposed method, experiments were performed using the data collected from Gyeongbu expressway. The results demonstrated that the proposed method could predict the traffic flow with an accuracy of 85%. Furthermore, its performance was compared with that of three existing methods, and the proposed method achieved a performance improvement of 113% compared with the existing method. 

Despite of its effectiveness in predicting the real-time traffic flow, the proposed method needs some improvements. (1) In this work, we employed only Gyeongbu expressway among 10 expressways in Korea. To fully demonstrate the effectiveness of our estimation model in prediction traffic flow, it should be tested on a variety of road conditions with high interactions. For this, we made an effort to obtain the road information from Korea expressway cooperation, and are scheduled to receive the whole expressway data from April 2016; (2) The noisy data have some adverse effects to the performance of traffic flow estimations; thus, it should be filtered. For this, the outlier detection that can find some abnormal data when comparing its neighbors on the spatiotemporal domain is required. In addition, such a method can be used for recognizing some seasonal effects and accidents; (3) To model the interaction between adjacent roads and between roads and their conditions, more clique parameters and model parameters should be required. To handle this complexity, we will employ the non-linear regression method such as a support vector regression (SVR) as well as linear regression. To improve the performance of the proposed method, we are under working on outlier detection and non-linear regression. 
